# Secreted Protein Acidic and Rich in Cysteine (*SPARC*)—Mediated Exercise Effects: Illustrative Molecular Pathways against Various Diseases

**DOI:** 10.3390/diseases11010033

**Published:** 2023-02-13

**Authors:** Abdelaziz Ghanemi, Mayumi Yoshioka, Jonny St-Amand

**Affiliations:** 1 Department of Molecular Medicine, Faculty of Medicine, Laval University, Quebec, QC G1V 0A6, Canada; 2 Functional Genomics Laboratory, Endocrinology and Nephrology Axis, CHU de Québec-Université Laval Research Center, Quebec, QC G1V 4G2, Canada

**Keywords:** secreted protein acidic and rich in cysteine (*SPARC*), exercise, molecular pathways, diseases

## Abstract

The strong benefits of exercise, in addition to the development of both the therapeutic applications of physical activity and molecular biology tools, means that it has become very important to explore the underlying molecular patterns linking exercise and its induced phenotypic changes. Within this context, secreted protein acidic and rich in cysteine (*SPARC*) has been characterized as an exercise-induced protein that would mediate and induce some important effects of exercise. Herein, we suggest some underlying pathways to explain such *SPARC*-induced exercise-like effects. Such mechanistic mapping would not only allow us to understand the molecular processes of exercise and *SPARC* effects but would also highlight the potential to develop novel molecular therapies. These therapies would be based on mimicking the exercise benefits via either introducing *SPARC* or pharmacologically targeting the *SPARC*-related pathways to produce exercise-like effects. This is of a particular importance for those who do not have the ability to perform the required physical activity due to disabilities or diseases. The main objective of this work is to highlight selected potential therapeutic applications deriving from *SPARC* properties that have been reported in various publications.

## 1. Secreted Protein Acidic and Rich in Cysteine (*SPARC*): An Exercise-Induced Biomolecule

Beyond being a social activity or a hobby, strong evidence has linked exercise to a variety of health benefits and has made physical activity a part of different therapies including for the treatment of obesity [1,2,3], diabetes [4], depression [5,6] anxiety [5,7], Parkinson disease [8], Alzheimer’s disease [9], Coronary heart disease [10], ageing and sarcopenia [11,12,13]. To reveal the mechanisms beyond the exercise benefits there was a need to explore the molecular and cellular changes underlying the exercise-induced changes. As genes are important factors of biomolecular and biochemical pathways, the changes in gene expression in response to exercise have been explored. Within this context, functional genomics has identified genes that are overexpressed with exercise [14,15]. Exploring these genes represents a significant starting point towards the mechanistic understanding of exercise. 

The most important of these gene expressions would be the secreted protein acidic and rich in cysteine (*SPARC*) [16]. Following the identification of *Sparc* as an exercise-induced gene (induced during endurance training), and as *SPARC* expression is also known to decline with age [17], *SPARC* implications have been explored in the contexts of both exercise and ageing. Briefly, studies using genetically-modified mice suggested that exercise-induced muscle phenotype changes are *SPARC*-dependent [18] and showed that *SPARC* overexpression mimics exercise effects in mice, whereas *Sparc* KO leads to an accelerated ageing phenotype which is improved by exercise [19]. Together, these data suggest that at least a part of the exercise benefits are mediated by *SPARC*, which would be anti-aging, and with effects against various metabolic disorders and age-related diseases [20,21]. *SPARC* is expressed in various situations and has even been suggested as a molecular physiological and pathological biomarker [22] for which its measure could optimize personalized medicine [23]. Herein, we suggest literature-based mechanisms to explain the exercise effects, the *SPARC* effects, as well as the molecular pathways beyond the exercise-induced effects that are mediated by *SPARC*, which is considered as an exercise-induced protein.

## 2. Related Pathological Concepts

Exercise is known for its benefits in enhancing metabolic functions and body fitness and for improving many risk factors including obesity, body fat, metabolic syndrome, lipidic profiles and insulin resistance [15,16]. These benefits correlate with exercise-induced gene expression regulation that include, for instance, increase in peroxisome proliferator-activated receptor gamma (PPARγ), coactivator 1-alpha (PGC1α), metabolic-related (TCA cycle, β-oxidation, electron transport and oxidative phosphorylation), antioxidant enzymes and contractile apparatus-encoded genes [16,24] among other genes [15]. Moreover, some exercise benefits such as those related to the improvement of muscular oxidative phosphorylation, calcium signalling, and tissue development have been found to remain even following training cessation [25]. The global trend towards physical inactivity has driven a dramatic increase in the incidence of many chronic diseases such as obesity, type 2 diabetes (T2D), hypertension, cardiovascular diseases (CVD), immune dysfunction, certain types of cancer, pulmonary diseases, musculoskeletal diseases and several types of neurodegenerative disorders [26].

Aging, defined as a chronic biological process of progressive functional decline in intrinsic physiological functions [14,27,28], also leads to chronic diseases, thus increasing the age-specific mortality rate [29]. The skeletal muscles are among the most age-sensitive tissues. Sarcopenia, a gradual loss of muscle mass, strength and function due to aging [30], is associated with reduced muscle regenerative capacity, mitochondrial dysfunction [31] and muscle fiber atrophy [31,32,33]. The most evident metabolic explanation is an imbalance between protein synthesis and breakdown rates. However, other causes include neurodegenerative processes, the reduction in anabolic hormone productions or sensitivity such as insulin, growth hormones and sex hormones, the dysregulation of cytokine secretions, modification in the response to inflammatory events, inadequate nutritional intakes and sedentary lifestyle [34,35,36].

Sarcopenia represents not only a risk factor for falling, a decrease of independence, and disability caused by immobility, but also for metabolic disorders, such as T2D and obesity [37,38]. Due to the skeletal muscle constituting the largest insulin-sensitive tissue in the body [39], and being the primary site for insulin-stimulated glucose utilization [39], T2D can be a consequence of muscle atrophy. Moreover, as skeletal muscle accounts for 40–50% of body weight and 20–30% of total resting energy expenditure [40], obesity can result from accumulated minor imbalances of energy intake over expenditure [41]. Under the obese status, the adipose tissue macrophages are a prominent source of the proinflammatory cytokines such as tumour necrosis factor α (TNF-α) and interleukin 6 (IL-6), both which can block the tissue insulin action and cause systemic insulin resistance, thus providing a potential link between inflammation and insulin resistance [42]. In the case of the etiology of atherosclerosis, the inflammation also plays a central role in developing CVD [43].

In addition, mitochondrial dysfunction resulting from oxidative damage to the mitochondrial DNA (mtDNA) caused by the reactive oxygen species (ROS) is one of the factors driving the aging process [44,45]. In an autoimmune disease, mitochondrial dysfunctions increase the ROS production that drive type I interferon-inducible gene expression and muscle inflammation [46]. The excessive ROS production can trigger mitochondrial dysfunction, apoptosis, autophagy, inflammation and muscle atrophy [47,48,49,50]. Moreover, mice lacking the cytoplasmic antioxidant enzyme, superoxide dismutase (SOD1), showed increased oxidative damage to proteins, lipids and DNAs, resulting in progressive muscle denervation, weakness and loss [51]. Thus, accumulation of the mtDNA mutations have also been linked to the pathogenesis of sarcopenia [52].

## 3. *SPARC*-Mediated Effects among the Exercise Benefits

**Figure 1:**
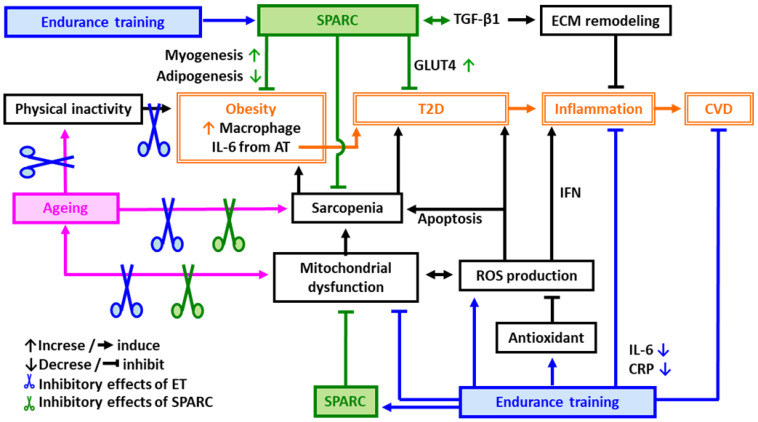
Possible mechanisms of protective effects of endurance training (ET) and *SPARC* against age-related metabolic disorders. **Abbreviations**: **AT**: adipose tissue, **CVD**: cardiovascular diseases, **CRP**: C-reactive protein, **ECM**: extracellular matrix, **ET**: endurance training, **GLUT4**: glucose transporter type 4, IL-6: interleukin 6, **IFN**: interferon, **ROS**: reactive oxygen species, ***SPARC***: secreted protein acidic and rich in cysteine, **T2D**: type 2 diabetes, **TGF-β1**: transforming growth factor beta 1.

Exercise is significantly superior to all known pharmacological, nutritional and hormonal interventions for stabilizing and reversing sarcopenia [53]. Endurance training (ET) is well known to activate the mitochondrial biogenesis/function and to reduce serum inflammatory mediators such as C-reactive protein (CRP) and IL-6 [54] that are both impaired with ageing [26,55]. The improved mitochondrial function/systemic inflammation ameliorates insulin sensitivity and the lipid profile [16,56,57], and contributes to a decrease in mortality rates [58]. Thus, ET improves both the anti-oxidative and the anti-inflammatory response in addition to ameliorating obesity, CVD risks and sarcopenia (Figure 1).

Physical inactivity leads to obesity and T2D resulting in an acceleration of inflammation. Inflammation plays a central role in developing CVD. ET is a key factor to prevent age-related metabolic disorders such as obesity, T2D and CVD through the improvement of mitochondrial function and sarcopenia as well as through the induction of antioxidants that eliminate the ROS and decrease apoptosis. *SPARC* also improves mitochondrial function, sarcopenia, obesity, T2D, CVD and inflammation.

**Figure 2:**
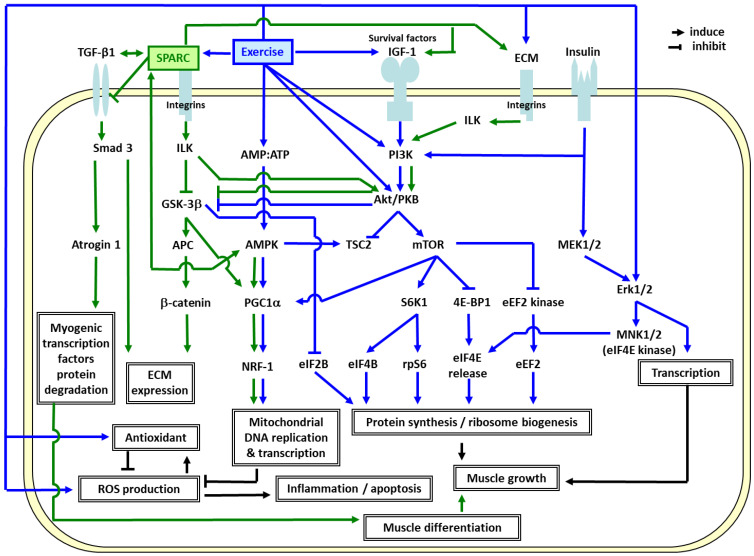
Possible mechanisms linking extracellular matrix (ECM), mitochondrial biogenesis, the effects of *SPARC* and exercise training. **Abbreviations**: **4E-BP1**: eIF4E-binding protein 1, **Akt/PKB**: RAC-alpha serine/threonine-protein kinase/protein kinase B, **AMP**: adenosine monophosphate, **AMPK**: AMP-activated protein kinase, **APC**: adenomatous polyposis coli, **ATP**: adenosine triphosphate, **CL-1**: collagenase 1/matrix metalloproteinase 1, **Dvl**: dishevelled, dsh homolog 1 (Drosophila), **ECM**: extracellular matrix, **eEF2B**: eukaryotic translation elongation factor 2B, **eIF2B/4B/4E**: eukaryotic translation initiation factor 2B/4B/4E, **Erk1/2**: extracellular-signal-regulated kinases 1/2, **FAK**: focal adhesion kinase, **GSK-3β**: glycogen synthase kinase 3 beta, **IGF-1**: insulin-like growth factor 1, **ILK**: integrin-linked kinase, **MEK1/2**: mitogen-activated protein (MAP) kinase kinase 1/2, **MNK1/2**: MAP kinase-interacting kinase 1/2, **mTOR**: mammalian target of rapamycin, **NRF-1**: regulating nuclear respiratory factor 1, **PGC1α**: peroxisome proliferator-activated receptor gamma coactivator-1α, **PI3K**: phosphoinositide 3-kinase, **ROS**: reactive oxygen species, **rpS6**: ribosomal protein S6, **S6K1**: ribosomal protein S6 kinase beta-1, **Smad3**: SMAD family member 3, ***SPARC***: secreted protein acidic and rich in cysteine, **TGF-β1**: transforming growth factor beta 1, **TSC2**: tuberous sclerosis protein 2.

In order to elucidate the molecular mechanisms responsible for the ET effects [14,28], we identified the genes specifically modulated by ET in elderly muscle compared to young adults, and highlighted the importance of mitochondrial oxidative phosphorylation (OXPHOS) and extracellular matrix (ECM) remodeling in the skeletal muscle [16,24,57,59]. As shown in Figure 2, the ET-induced genes in elderly men [16,24,57,59], *SPARC*, specifically binds several of the ECM molecules including the collagens [60]. Thus, they influence lamina organization by binding to the growth factors such as insulin-like growth factor 1 (IGF-1) and transforming growth factor beta 1 (TGF-β1) [61,62,63]. TGF-β1 (profibrotic and anti-inflammatory protein) induces *SPARC* expression and vice versa [64]. While the extracellular *SPARC* functions as a matricellular protein, the intracellular and membrane-associated *SPARC* regulates cellular apoptotic pathways [64].

After myocardial infarction, *SPARC* is expressed by inflammatory cells [65], suggesting that *SPARC* produced by infiltrating leukocytes has a role in the inflammatory response and fibrosis in the heart. Thus, the absence of *SPARC* results in increased cardiac rupture and dysfunction [65]. By facilitating monocyte recruitment and/or macrophage differentiation and tissue retention [66], *SPARC* and TGF-β1 may be involved in minimizing inflammation [67]. *SPARC* also functions in the production and remodeling of the adipose tissue as well as in the regulation of preadipocytes differentiation [68]. In the absence of *SPARC*, mice show enhanced diet-induced obesity [68]. Furthermore, we have shown that *SPARC* increases type I collagen and OXPHOS expressions in proliferating and differentiating myoblasts in addition to accelerating differentiation, whereas inhibition leads to the opposite effects [69]. Moreover, we have confirmed an induction of myokine, *Sparc*, and PGC1α expressions in myoblasts after 48 h of electrical pulse stimulation, which is a suitable exercise model in vitro [70].

The results suggest that the exercise-induced *SPARC* plays a crucial role in the muscle integrity through ECM remodeling and mitochondrial biogenesis. Figure 2 also illustrates how ET promotes muscle growth and mitochondrial biogenesis via IGF1-phosphoinositide 3 kinase (PI3K)-Akt-mammalian target of rapamycin (mTOR) [71,72,73,74,75,76,77,78,79] and AMP-activated protein kinase (AMPK)-PGC1α [80,81,82] pathways. Exercise-induced *SPARC* regulates ECM remodeling via integrin-linked kinase (ILK)-glycogen synthase kinase 3 beta (GSK 3β)-β catenin [83,84], and transforming growth factor beta 1 (TGF β1)-SMAD family member 3 (Smad3) pathways [85]. *SPARC* binds to TGF-β1 co-receptor and inhibits the binding of TGF-β1 to its receptor. Thus, the TGF β1-Smad3-atrogin 1 pathway, in turn suppresses myogenic transcription factors (Myo D and myogenin) degradation and promotes muscle differentiation [86]. *SPARC* also interacts with AMPK [70,87] which induces PGC1α [80] and stimulates the ILK-GSK3β-PGC1 pathway [88], which may lead to mitochondrial biogenesis through a powerful induction of regulating nuclear respiratory factor 1 (NRF1) [82]. ET also induces antioxidants which eliminate the ROS, and consequently decrease apoptosis and inflammation. Other metabolic effects of *SPARC* on the cells have been shown in murine in cultured 3T3-L1 white and HIB1B brown adipocytes and the results suggest that recombinant *SPARC* both activates brown adipocytes and upregulates white adipocytes browning [89].

In order to further clarify the in vivo roles of *SPARC* and their similarities with ET, the impacts of *Sparc* knock-out (KO) in relation to sarcopenia and age-related metabolic disorders in young and old mice have also been investigated [18,19,90]. As expected, aging and/or *Sparc* KO led to sarcopenia (decreased muscle mass and strength), decreased glucose tolerance, and decreased expressions of muscle glucose transporter type 4 (GLUT4), collagen and OXPHOS, whereas ET had the opposite effects. Such *Sparc* KO-induced phenotype is important to understand the roles of *SPARC* in sarcopenia and glucose metabolism and to understand the link between ECM remodeling and mitochondrial function. Overall, *SPARC* can mimic the effects of ET which include the modulation of the ECM, mitochondrial function, inflammation, the tissue integrity and the immune response, as well as myogenesis, adiposity and glucose homeostasis [18,69,70,90,91,92,93,94,95,96]. Therefore, *SPARC* would be a key molecular link between physical exercise, obesity, T2D, CVD and inflammation (Figure 1 and Figure 2).

Nevertheless, a true confirmation regarding the potential of *SPARC* to be an exercise surrogate would be the addition/introduction of *SPARC* into a biological system. Thus, transgenic (Tg) mice over-expressing *Sparc* gene were created, and compared to both *Sparc* KO mice and an ET-induced phenotype [19]. The young *Sparc* Tg mice had increased muscle strength, muscle mass, and muscle glucose transporter and OXPHOS expressions, but lower glycemia and adiposity, an effect especially found in males [19]. Collectively, these findings showed that *Sparc* KO mice manifested an aging-like phenotype, whereas *SPARC* overexpression and exercise generated similar benefits [19]. The benefits were in regard to counteracting both *SPARC* deficiency-induced aging-like phenotype in addition to reversing age-related changes [19]. Indeed, *Sparc* overexpression would counteract some of the aging effects, most likely by activating the ILK-ECM pathway [91] via *SPARC* induction, as well as the mTOR-protein synthesis pathway (Figure 2). The *Sparc* KO lowered the muscle mitochondrial OXPHOS proteins, whereas the aging effects were only seen in the WT mice, since their levels in young *Sparc* KO mice were already as low as the old WT mice [97]. Theoretically, *Sparc* overexpression and ET would counteract the aging effects, most likely by inducing the antioxidant enzymes and the mitochondrial biogenesis inducer, PGC1α (Figure 2).

Both ET and *Sparc* overexpression would counteract aging-related dyslipidemia and glucose intolerance (Figure 1). We expected that *SPARC* would be, in part, involved in the mechanisms mediating ET-induced skeletal muscle adaptation, which in turn, would improve age-related diseases. Muscle atrophy occurs when a balance between anabolism and catabolism shifts toward excessive catabolism. The ubiquitin-proteasome system is the main regulatory mechanism of protein degradation in the skeletal muscle. The muscle specific ubiquitin-ligase enzymes (E3s), the muscle RING finger-1 (MuRF1) and the muscle atrophy F-box (MAFbx, also known as atrogin 1), regulate skeletal muscle atrophy in various pathological and physiological conditions by inducing the degradations of the structural proteins such as myosin light chain 2 and troponin I as well as myogenic differentiation proteins such as MyoD and myogenin, respectively [98,99,100]. The loss of *SPARC* in the mouse skeletal muscle causes myofiber atrophy by enhancing TGF-β1 signaling via phosphorylation of Smad3. Thus, it upregulates the atrogin 1 expression which may, in turn, cause muscle atrophy [86]. In mice, hindlimb immobilization leads to a 36% reduction in myofiber size and to an early inflammatory process during atrophy [101]. Moreover, exercise training, prior to the immobilization, alleviates muscle atrophy [102]. Therefore, we also hypothesize that *SPARC* may optimize or act similarly to exercise in order to attenuate muscle atrophy, which, in turn, prevents sarcopenia and age-related diseases (Figure 1).

## 4. Perspectives and Significance

Understanding *SPARC* implications during various biological processes and its potential roles in preventing or treating different diseases and health conditions such as obesity, sarcopenia, ageing and metabolic disorders could lead to important therapeutic tools to deal with such challenging health problems. The *SPARC* properties can be of use for pharmaceutical companies to develop molecules that mimic *SPARC* or that target *SPARC*-related pathways to counteract those specific health problems. Among such health problems, obesity is a condition involving different factors (biochemistry [103], genetics [104], hormones [105], DNA damage [106], etc.) and it has developmental patterns that have even been compared to cancer [107].

Indeed, exploring the pathways described above would allow for a better understanding of how *SPARC* mediates exercise-induced benefits and would reveal the molecular pathways linking physical activity to its induced phenotype. Such mechanistic understanding would help to develop a new generation of molecular therapies that mimic exercise. The principle underlying these therapies would be to either administer *SPARC* or pharmacologically target *SPARC*-related pathways to generate exercise-like benefits. Within this context, the importance of other considerations such as the predictive analysis of the *SPARC* molecular structure (for instance via AlphaFold [108]) could suggest potential mechanistic hypotheses for *SPARC* mechanisms of action.

Therapeutic applications can be speculated on based on the various functions and properties that *SPARC* has been associated with such as anti-inflammatory [94], regeneration [92], anticancer [109] and metabolism [110,111]. Further studies are required to investigate whether systemic injection or expression is the best option, or whether it is the targeting of specific tissues that would improve the outcomes depending on the targeted health problems.

## Data Availability

Not applicable.
